# West Nile Virus Detection in Kidney, Cloacal, and Nasopharyngeal Specimens

**DOI:** 10.3201/eid1109.050016

**Published:** 2005-09

**Authors:** Ojimadu A. Ohajuruka, Richard L. Berry, Sheila Grimes, Susanne Farkas

**Affiliations:** * Ohio Department of Health, Columbus, Ohio, USA;; † Ohio Department of Agriculture, Columbus, Ohio, USA

**Keywords:** West Nile virus, TaqMan RT-PCR, crow, blue jay, cloacal swab, threshold value, surveillance, correlation, linear regression, dispatch

## Abstract

We compared kidney tissue samples and cloacal and nasopharyngeal swab samples from field-collected dead crows and blue jays for West Nile virus surveillance. Compared to tissue samples, 35% more swab samples were false negative. Swab samples were usually positive only when the corresponding tissue sample was strongly positive.

Monitoring and surveillance of West Nile virus (WNV) prevalence increasingly depends on the early detection of WNV infection in crows, blue jays, and other members of the avian family *Corvidae* on the basis of reports of dead birds. The virus in these birds usually precedes appearance of WNV in humans and can be an early warning of potential human infection (*1**–**3*). After the initial finding of WNV in the United States in 1999, various laboratory techniques have been developed and employed to detect WNV in avian tissue. A WNV-specific RNA assay by TaqMan reverse transcriptase polymerase chain reaction (RT-PCR) has been described (*4*). This protocol has gained widespread recognition because of its high specificity, sensitivity, and speed. The sensitivity of the TaqMan RT-PCR is equal to, or better than, that of the Vero plaque assay method (*5*). WNV activity has increased in the United States since 1999; various approaches are being used to fully understand and appropriately respond to the infection (*6*).

WNV can be detected in a wide variety of bird tissue, such as heart, liver, lung, and spleen. The kidney also provides good specimen material for WNV detection (*5*). Brain tissue is the most sensitive target organ for detecting WNV with the TaqMan RT-PCR assay. In Ohio, where 31 persons died of WNV in 2002, kidney tissue was removed from >2,500 American crows (*Corvus brachyrhynchos*) and blue jays (*Cynocitta cristata*) to test for WNV. These birds are among the most susceptible species to WNV infection and have high death rates. For field-collected avian samples in Ohio, kidney tissue has been the sample of choice to detect WNV by using RT-PCR, mainly because of the practical ease and convenience of sampling kidney tissue specimens compared to brain tissue specimens.

We examined the suitability of using other specimens that are easier to collect because harvesting tissue invasively is labor intensive, the chance of cross contamination of samples will be minimized, and the possibility of exposing laboratory workers to infection will be reduced. Some researchers hypothesized that cloacal and oral swabs from avian carcasses could replace brain samples, the preferred tissue to test for WNV infection in corvid carcasses (*5**,**7*). In this study, we compared the suitability of testing kidney tissue and cloacal and nasopharyngeal swab specimens from field-collected dead birds to detect WNV in crows and blue jays.

## The Study

During the 2002 WNV surveillance season, dead crows and blue jays were collected throughout Ohio. The dead birds were wrapped in plastic bags and hand-delivered or shipped in refrigerated containers to the Animal Disease Diagnostic Laboratory of the Ohio Department of Agriculture. In the laboratory, the cloacal and nasopharyngeal areas were swabbed from each bird individually with standard cotton applicators. The swab from each dead bird tissue was put into a separate prelabeled 12 × 75 mm tube containing 0.5 mL BA-1 medium (M-199 salts, 1.0% bovine serum albumin, 350 mg/L sodium bicarbonate, 100 units/mL penicillin, 100 mg/L streptomycin, and 1.0 mg/L amphotericin in 0.05 mol/L Tris [hydroxymethyl aminomethane], pH 7.6). The kidneys of each of these birds were harvested after evisceration, and specimen samples were put into individual vials. The time of death of the field-collected crows and blue jays could not be ascertained by the collectors but was generally believed to be within 48 hours postmortem. Decomposed carcasses were not accepted for testing. The kidney tissue specimens and cloacal swab and nasopharyngeal swab specimens from the dead birds were stored at –70°C until tested.

To test avian kidneys for WNV with RT-PCR, ≈0.4 g of kidney sample from each bird was homogenized in a prelabeled, snap-cap vial containing 0.5 mL BA-1 medium and 2 ball bearing caliber air gun shot pellets. Vials for processing were centrifuged, and 75 μL of the supernatant from each sample was used for subsequent viral RNA extraction and purification by using the QIAamp Viral RNA minikit according to the manufacturer's recommended protocol (Qiagen, Valencia, CA, USA). The RNA extracts were assayed by a TaqMan RT-PCR with a TaqMan reverse transcriptase-PCR kit (Applied Biosystems, Foster City, CA, USA). For the TaqMan assay, primers and probes with the following nucleotide sequences (5´–3´) as previously described by Lanciotti et al. (2000) were used: forward primer CAGACCACGCTACGGCG, reverse primer CTAGGGCCGCGTGGG, and FAM/TAMRA probe TCTGCGGAGAGTGCAGTCTGCGAT. Thermal cycling was performed with the Bio-Rad i-Cycler iQ Real-Time detection system (Bio-Rad Laboratories, Hercules, CA, USA). At the end of the reaction, the amplification plot generated was viewed on a log scale with the system's default threshold. Any sample with a threshold cycle (C_T_) of <35 was considered to be positive for WNV. This value corresponds to the detection of >1–10 PFU in WNV-infected specimens (*4*).

After the RT-PCR preliminary assays on the kidney samples were conducted, 100 of the avian kidney samples that had tested positive for WNV were randomly selected for further investigation; 61 of the samples were from crows and 39 were from blue jays. The corresponding 100 cloacal and 100 nasopharyngeal swab samples of these positive birds were additionally tested for WNV. In the test, each cloacal or nasopharyngeal swab sample was thoroughly mixed in the BA-1 medium by vortexing. As with the kidney samples, a 75.0-μL aliquot of this medium was used for viral RNA extraction and purification, as well as RT-PCR amplification as described above. Statistical analyses of the results from the tests were performed by using the SPSS for Windows release 10.0.1 (SPSS Inc., Chicago, IL, USA) to analyze the data.

The 100 positive kidney samples had various C_T_ values (the cycle at which the fluorescence rises appreciably above the background level), which ranged between 15.9 and 35.0. When all of the 100 positive kidney samples were divided into 3 categories of "high" (C_T_ 15.0–21.9), "medium" (C_T_ 22.0–28.9), or "low" (C_T_ 29.0–35.0) positive results based on their threshold cycle values, 57.0%, 27.0%, and 16.0% of the kidney samples fell into these respective positive groups (Table). Because no meaningful difference was seen between the test results of the crows and the blue jays in these categories, the data from both bird species were combined in our analyses. None of the cloacal or nasopharyngeal swab samples were in the high-positive group. Seventy-seven percent of the cloacal and nasopharyngeal swab samples were either in the low-positive or negative categories. The mean C_T_ value of the kidney samples was <0.01 lower than those of the cloacal and nasopharyngeal samples. Contrary to earlier findings (*8*) in which oropharyngeal swabs were more sensitive than cloacal swabs by using the VecTest antigen-capture assay, we found no appreciable difference in our study between the C_T_ values of the cloacal and nasopharyngeal swab samples.

**Table:** Percentage of kidney, cloacal, and nasopharyngeal swab samples with high, medium, and low positive or negative RT-PCR results (N = 100)*

Value	High positive	Medium positive	Low positive	Total positive	Total negative
Threshold cycle (C_T_) range	15.0–21.9	22.0–28.9	29.0–35.0	C_T_<35.0	C_T_>35.0
Kidney tissues, %	57	27	16	100	0
Cloacal swabs, %	0	23	41	64	36
Nasopharyngeal swabs, %	0	23	43	66	34

*RT-PCR, reverse transcription–polymerase chain reaction..

A positive correlation was seen between the kidney test results and both cloacal and nasopharyngeal swab samples (R^2^ = 0.62 and 0.53, respectively, Figure). The correlation and linear regression analyses indicate that both the cloacal and nasopharyngeal swab samples showed a smaller proportion of the positive specimen than did the kidney testing. Although viral amounts in the samples were not quantified, virus was detected in the cloacal and nasopharyngeal samples, usually only when the viral load in the kidney samples from the birds was high.

**Figure:**
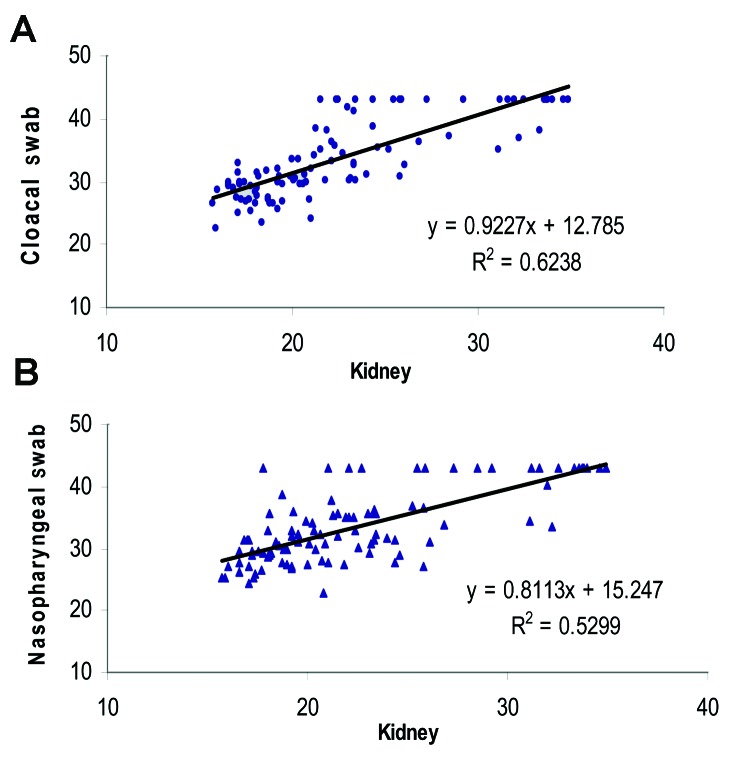
Linear regression plots of avian kidney C_T_ values versus A) cloacal and B) nasopharyngeal C_T_ values using the linear regression model Y´i = bX´i + a.

## Conclusions

The brain is reportedly the most sensitive organ to detect WNV in American crows (*5*). Other internal organs such as liver, kidney, spleen, heart, and lungs are also useful to detect viruses. However, because these organs can only be obtained through necropsy, easier, but equally sensitive, methods of sample collection are needed. Earlier research results on the value of testing cloacal and nasopharyngeal samples have not been consistent, reflecting differences in sampling methods. WNV was detected in all 20 postmortem brain tissue samples as well as cloacal and oral swabs of crows and blue jays that were experimentally infected with >10^5^ PFU WNV (*7*). High viral infections in the bird kidney result in positive swab specimens, however, lower viral amounts may not.

We did not determine if the reduced amount of viral RNA in the swab samples could have been due to inactivation during the 4 months of storage after sampling. Cloacal swabs were less sensitive than oropharyngeal swabs to detect WNV (*8*). Kidney tissue samples that were ground in BSA-1 solution and stored at –70°C retained their sensitivities in subsequent tests for >2 years. Under the field conditions of our study, differences in the time of death, environmental conditions, and stage of virus spread in the bird, may have influenced the results.

The brain, in particular the cerebellum, was a primary target of infection in birds with WNV (*9*). Several studies have found the testing of avian kidneys to provide sufficient information on WNV infectivity in birds (*5**,**8*). The sharper focus of this study was to determine testing methods in which it would be easy to extract samples from the birds. However, these findings do provide an insight into expected outcomes if cloacal or nasopharyngeal swab samples, rather than kidney tissue samples, are submitted to test for WNV with TaqMan RT-PCR. The linear regression equations provide a predictive value of determining the C_T_ value of cloacal and nasopharyngeal swab samples; however, the swab samples usually show positive results only when the viral load in the kidney is high or strongly positive (low or medium C_T_ values).

When testing for WNV with TaqMan RT-PCR (*4*), substituting cloacal or nasopharyngeal swab samples for kidney tissue samples would result in ≈35% fewer positive field-collected samples. Testing cloacal or nasopharyngeal swab samples might, however, be useful in situations where underreporting of the positives may not be of concern, when evidence of infection is predominant in a locality, and where the ease of obtaining cloacal or nasopharyngeal swabs makes it an attractive choice to detect WNV in dead crows and blue jays. In practice, we believe that cloacal and nasopharyngeal swabs would be easier to perform in the field. Also, savings are related to lower shipping costs and eliminating necropsy procedures.
